# Synergetic Improvement of Stability and Conductivity of Hybrid Composites formed by PEDOT:PSS and SnO Nanoparticles

**DOI:** 10.3390/molecules25030695

**Published:** 2020-02-06

**Authors:** Antonio Vázquez-López, Anisa Yaseen, David Maestre, Julio Ramírez-Castellanos, Erik S. Marstein, Smagul Zh. Karazhanov, Ana Cremades

**Affiliations:** 1 Departamento de Física de Materiales, Facultad de CC. Físicas, Universidad Complutense de Madrid, 28040 Madrid, Spain; antvaz01@ucm.es (A.V.-L.); davidmaestre@fis.ucm.es (D.M.); cremades@fis.ucm.es (A.C.); 2 Department for Solar Energy, Institute for Energy Technology, 2027 Kjeller, Oslo, Norway; anisa.yaseen@fys.uio.no (A.Y.); Erik.Stensrud.Marstein@ife.no (E.S.M.); 3 Departamento de Química Inorgánica, Facultad de CC. Químicas, Universidad Complutense de Madrid, 28040 Madrid, Spain; jrcastel@quim.ucm.es

**Keywords:** SnO, PEDOT:PSS, hybrid composite

## Abstract

In this work, layered hybrid composites formed by tin oxide (SnO) nanoparticles synthesized by hydrolysis and poly(3,4-ethylenedioxythiophene)-poly(styrenesulfonate) (PEDOT:PSS) have been analyzed. Prior to the composite study, both SnO and PEDOT:PSS counterparts were characterized by diverse techniques, such as X-ray diffraction (XRD), Raman spectroscopy, transmission electron microscopy (TEM), photoluminescence (PL), atomic force microscopy (AFM), optical absorption and Hall effect measurements. Special attention was given to the study of the stability of the polymer under laser illumination, as well as the analysis of the SnO to SnO_2_ oxidation assisted by laser irradiation, for which different laser sources and neutral filters were employed. Synergetic effects were observed in the hybrid composite, as the addition of SnO nanoparticles improves the stability and electrical conductivity of the polymer, while the polymeric matrix in which the nanoparticles are embedded hinders formation of SnO_2_. Finally, the Si passivation behavior of the hybrid composites was studied.

## 1. Introduction

SnO is a p-type semiconducting oxide which has demonstrated promising applicability as an anode for Li-ion batteries [1], coating for solar energy [2], gas sensing [3], and thermoelectricity [4], among other fields of technological research. However, few works can be found focused on SnO-based applications due to its inherent metastability, as it can be easily oxidized to the most stable SnO_2_. Hence, the applicability of SnO is commonly hindered, regardless of its potential interest as a p-type material in optoelectronics and energy-related applications, based on its good electrical conductivity due to native Sn vacancies and its wide bandgap (2.7–3.4 eV). In order to overcome this limitation, in most cases SnO is encapsulated or combined with SnO_2_ [5,6] or other materials [7,8,9]. Fabrication of composites formed by SnO and organic materials such as carbonaceous compounds or polymers has also received increasing interest in recent years. Hybrid composites combining organic and inorganic materials have recently become of great interest in fields such as sensors [10], and in microelectronic devices, based on the synergy between their counterparts which can lead to the development of flexible, low-cost, and scalable devices with good stability and tunable electrical and optical properties. In particular, special interest has been recently aroused by poly(3,4-ethylenedioxythiophene)-poly(styrenesulfonate) (PEDOT:PSS) in photovoltaic applications [11], as addition of inorganic materials allows to achieve new functionalities and improves the performance [12] of technologies such as hybrid silicon solar cells [13]. However, despite the promising performance reported for hybrid composites so far, several aspects still need to be further addressed, to overcome some limitations of these compounds and improve their applicability. In particular, most of the organic polymers used in composites lack stability, which also limits their applicability.

Poly(3,4-ethylenedi-oxythiophene) doped with polystyrenesulfonate (PEDOT:PSS) has been extensively used in organic electro-optical devices manly due to its transparency in the visible range and good p-type electrical conductivity. This polymer has demonstrated potential applicability in thermoelectric devices [14], Li-ion batteries [15], sensors [16], and other electronic devices [17]. However PEDOT:PSS shows a relatively low conductivity, typically in the range of 0.1–1 S cm^−1^, which can be improved by adding appropriate additives such as sorbitol, glycerol, or polar solvents including Dimethyl sulfoxide (DMSO) [18], Ethylene glycol (EG), and dimethylformamide (DMF), among others [19]. Such additives can improve the PEDOT:PSS conductivity up to two orders of magnitude, mainly due to changes on the PEDOT:PSS chains’ structure configuration [18]. The combination of PEDOT:PSS with inorganic nanomaterials can lead to improved performance and tunable optoelectronic properties [20]. As an example, the addition of nanoparticles can modify the Coulombic interaction of positively charged PEDOT and negatively charged PSS, thus obtaining better electrical transfer [21]. In this work we investigate the synergetic enhancement of the conductivity and stability of PEDOT:PSS by adding SnO nanoparticles in a controlled ratio, together with the hindering of the oxidation of the metastable SnO by the polymer encapsulation. Moreover, changes in the optical absorption of the polymer and the silicon substrate surface passivation have been evaluated, which could arouse potential interest in the photovoltaic field of research.

In the present work, hybrid composites formed by SnO nanoparticles combined in a controlled ratio with a conductive polymer PEDOT:PSS were synthesized. The hybrid layers were spin coated onto Si or glass substrates for their study. SnO nanoparticles, PEDOT:PSS, and hybrid composites were characterized by X-ray diffraction (XRD), transmission electron microscopy (TEM), selected area electron diffraction (SAED), Raman spectroscopy, photoluminescence (PL), atomic force microscopy (AFM), optical absorption, Hall effect measurements, and quasi steady state photoconductance (QSS-PC).

Hereafter, the samples are named as *SnO* (nanoparticles), *PEDOT:PSS* (polymer with DMSO and Triton X-100), and *Composite* (PEDOT:PSS with DMSO, Triton X-100, and SnO 1% wt.).

## 2. Results and Discussion

### 2.1. SnO Nanoparticles

**Figure 1:**
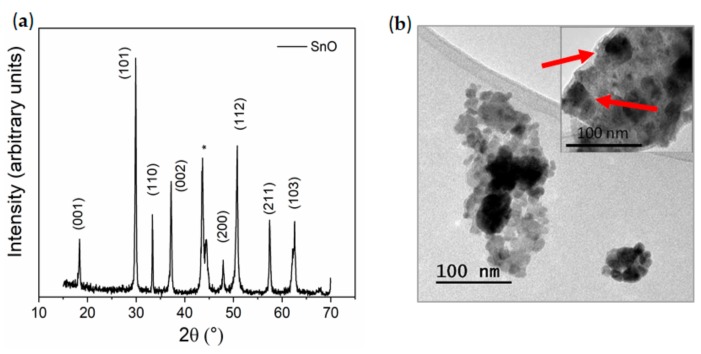
(**a**) XRD pattern and (**b**) TEM micrographs from SnO nanoparticles. The inset in (**b**) shows some nanoplates, marked with arrows. The peak marked with an asterisk in (**a**) corresponds to the sample holder.

X-ray diffraction results confirmed that samples can be indexed based on the romarchite SnO structure (ICDS 01-072-1012) with tetragonal structure, as shown in Figure 1a. No secondary phases were observed in this case. SnO nanoparticles show high purity and crystallinity. According to the Scherrer formula, an averaged crystallite size around 29 nm was estimated from the XRD analysis. TEM images confirm the presence of rounded SnO nanoparticles with dimensions around 10–20 nm (Figure 1b). In addition, SnO nanoplates with dimensions from 20 to 50 nm can also be observed (marked with arrows in the inset in Figure 1b), although in a lower concentration than the nanoparticles (inset in Figure 1b). Similar 2D nanostructures in forms of plates or flakes have been reported for SnO [22,23].

**Figure 2:**
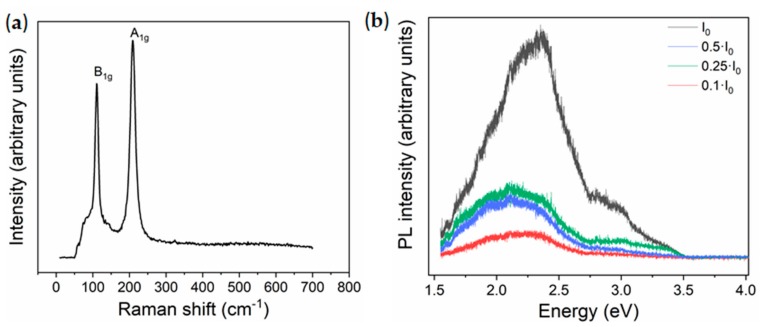
(**a**) Raman spectrum acquired with the He-Ne (633 nm) laser; (**b**) photoluminescence (PL) spectra acquired with the UV laser (325 nm) and variable laser intensity from SnO nanoparticles.

Raman spectrum from the SnO nanoparticles (Figure 2a) was acquired using the He-Ne laser (633 nm) and low power density in order to prevent possible formation of SnO_2_ during the analysis. Two main peaks centered at 110.5 cm^−1^ and 208.6 cm^−1^ can be clearly distinguished, which can be assigned to the characteristic B_1g_ and A_1g_ vibrational modes from SnO, respectively [24,25]. Peaks from SnO_2_ are not detected in the Raman spectra, within the resolution of the technique, in agreement with XRD measurements. The Raman analysis confirms not only high SnO purity but also that transition to SnO_2_ is avoided when using the He-Ne (633 nm) laser. On the other hand, by irradiation with the UV laser (325 nm), peaks from SnO_2_ are clearly observed in the Raman spectra at 460 cm^−1^ (E_g_) and 640 cm^−1^ (A_1g_) (Figure S1) due to the laser-induced transition from SnO to SnO_2._ Phase transitions achieved by appropriate laser irradiation have also been reported for other oxides [26], hence these aspects should be taken into account not only for the unambiguous analysis of the Raman signal, but also to either prevent or promote the transition by appropriate irradiation conditions.

As SnO exhibits a direct optical bandgap of 2.7 eV [27], together with an indirect bandgap of 0.7 eV, photoluminescence measurements were performed using a UV laser (325 nm). However, irradiation with the UV laser induces transition to SnO_2_, as confirmed by Raman analysis, hence neutral filters were used during the PL analysis in order to reduce the laser power density. All the PL spectra in Figure 2b show a wide emission in the visible range centered between 2 and 2.5 eV. The PL signal acquired with the lowest laser intensity corresponds to a wide band from 1.5 to 2.7 eV. For the PL spectrum acquired with the highest laser intensity, the dominant emission is centered at around 2.5 eV. Additionally, a shoulder at about 3 eV, followed by a weak tail up to 3.5 eV can be observed. Different authors reported emissions from SnO around 2 to 2.5 eV due to defects such as Sn vacancies and O vacancies, and at ~3 eV related to band-edge emissions [28]. SnO_2_ also shows characteristic emissions at 1.94 eV and 2.25 eV associated with oxygen vacancies-related defects, 2.58 eV due to surface defect states [29], and ~3 eV due to transitions involving Vo’’ levels [30]. All these bands can be present in the spectra shown in Figure 2b, hence in this case the study of the PL signal is not straightforward and careful attention should be paid during the analysis, as contributions from both SnO and SnO_2_ can be considered. However, considering that high, intense UV illumination promotes the formation of SnO_2_, the emissions in the high energy range, of which relative intensity is enhanced by using high laser power density, could be attributed to the increased presence of SnO_2_.

### 2.2. PEDOT:PSS Polymer

**Figure 3:**
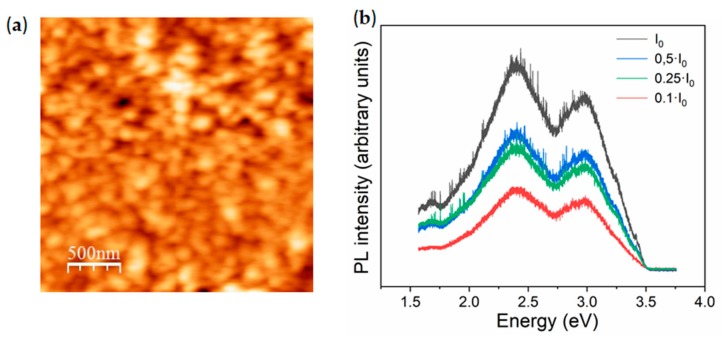
(**a**) Atomic force microscopy (AFM) image; (**b**) PL spectra acquired with the UV laser (λ = 325 nm) and variable laser intensity from spin-coated poly(3,4-ethylenedioxythiophene)-poly(styrenesulfonate) (PEDOT:PSS).

Bare PEDOT:PSS films deposited by spin coating onto Si or glass substrate have also been studied. AFM measurements indicate that the spin-coated PEDOT:PSS films onto Si substrates show smooth surfaces, with an average surface roughness of 3.5 ± 0.5 nm, formed by grains tens of nm in length (Figure 3a). The thickness of the spin-coated film is around 120 nm, as measured by AFM (not shown here). Differences on the AFM contrast are commonly associated with changes in the surface topography, however some authors propose that AFM contrast in PEDOT:PSS can be due to differences between PEDOT rich zones (bright areas) and PSS rich zones (dark areas) [31].

Photoluminescence from bare PEDOT:PSS was studied using a UV laser (λ = 325 nm) as the excitation source (Figure 3b). In this case, the PL spectrum acquired with the highest laser intensity shows a broad emission in the visible range formed by bands at 2.4 eV and 3.1 eV. Photoluminescence from PEDOT:PSS has not been extensively investigated so far, hence controversy still remains regarding the identification of the PL emission. Koyama et al. [32] attributed PSS to be responsible for PL emission from 1.2 to 3 eV. Some other authors [21] proposed emissions around 3 eV are due to PSS chains. In this case, higher laser intensity irradiation promotes an increase in the total PL signal, together with a slight increase in the relative intensity of the emission at 2.4 eV. Extended irradiation time tends to decrease the total intensity of the PL emission from the polymer, which should be due to its low stability under UV laser irradiation. PL measurements were also acquired on the bare Si substrate, in order to consider possible contributions to the PL signal related to the substrate onto which the PEDOT:PSS layer was spin coated. No PL emissions were detected in that case, thus confirming that the PL signal shown in Figure 3b is related to the analyzed polymer.

**Figure 4:**
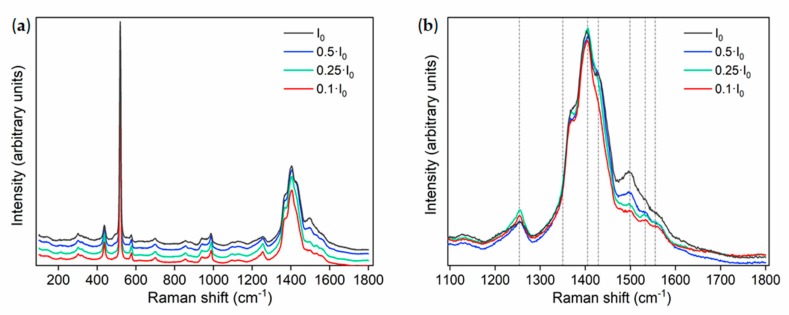
(**a**) Raman spectra acquired on PEDOT:PSS using the UV laser and variable irradiation conditions; (**b**) enlarged region marked in (a) corresponding to the 1100–1800 cm^−1^ range.

Raman spectroscopy was employed not only for the PEDOT:PSS characterization but also to study its stability under laser irradiation. Figure 4 shows the Raman spectra from bare PEDOT:PSS deposited on Si, acquired with the UV laser (λ = 325 nm) using different neutral filters in order to attenuate the initial laser power density. Main Raman vibrational modes from PEDOT:PSS are placed at around 1254 cm^−1^ related to C-C interring stretching [33], 1366 cm^−1^ due to single C_β_-C_β_ stretching [34,35], 1400 cm^−1^ associated with C_α_=C_β_, 1429 cm^−1^ due to C_α_=C_β_ symmetric stretching vibrations, and 1500 and 1561 cm^−1^ due to C_α_=C_β_ asymmetric stretching vibration related to the carbons on the thiophone ring in PEDOT in the middle and the end of the chains, respectively [36]. The peak around 1530 cm^−1^ arises from the splitting of the C_α_=C_β_ asymmetric vibrations [36]. The vibrational modes around 1400 cm^−1^ exhibit the higher relative intensity in all the Raman spectra, apart from the peak from the Si substrate centered at 520 cm^−1^. In this case, bare PEDOT:PSS shows good stability under UV laser illumination, however slight changes are observed in the Raman signal when high laser power density was used, as observed in Figure 4b. Higher laser intensity induces an increase in the relative intensity of the Raman modes at 1500 cm^−1^ and to a lower extent at 1429 cm^−1^. Some authors proposed changes in this region are due to laser-induced variation from benzoid to quinoid form [36], and the splitting of asymmetrical C_α_=C_β_ stretching vibrations in the polymer [34]. The relative intensity of the vibrational mode related to C-C interring stretching, centered at 1254 cm^−1^, decreases for high power density irradiation. These effects indicate small, structural changes in PEDOT:PSS promoted under high power UV illumination. It should be remarked that these structural changes under UV laser irradiation are enhanced in thinner PEDOT:PSS films, as expected (not shown here).

### 2.3. Hybrid Composite (SnO and PEDOT:PSS)

**Figure 5:**
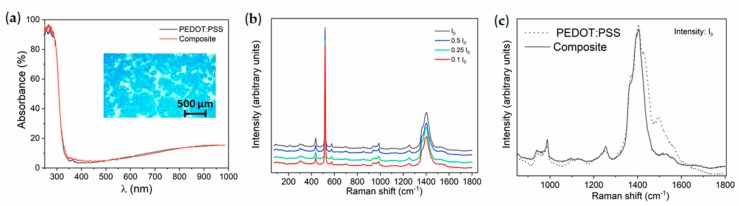
(**a**) Absorption spectra acquired on PEDOT:PSS and hybrid composites, with an optical image from the hybrid composite included in the inset; (**b**) Raman spectra from the hybrid composite acquired with variable UV laser intensity; (**c**) Raman spectra for bare PEDOT:PSS and the hybrid composite acquired using the same laser intensity (I_0_).

The hybrid composite shows good transparency in the visible range and slight improvement of the absorption in the range 350–450 nm, as compared with bare PEDOT:PSS, due to the addition of SnO nanoparticles (Figure 5a). The wide optical bandgap from SnO can explain the slightly improved absorption in the hybrid composite. An optical image from the hybrid composite is shown in the inset in Figure 5a, where good homogeneity and dispersion of SnO can be appreciated.

Raman spectra from the hybrid composite acquired with the UV laser and variable laser intensity are shown in Figure 5b. In this case, no remarkable variations in the Raman signal are observed as a function of the UV laser intensity, contrary to the changes described for bare PEDOT:PSS (Figure 4), which demonstrates improved stability of the polymer under UV illumination by adding SnO nanoparticles. Peaks from SnO_2_ were not observed after UV illumination, which can be related to the fact that the PEDOT:PSS matrix prevents SnO oxidation to SnO_2_. The addition of SnO nanoparticles, even in a low concentration (1% wt.), induces slight variations in the Raman signal from the hybrid composite in comparison with bare PEDOT:PSS, as shown in Figure 5c. Some other authors also reported changes in the properties of the polymer by adding low amounts of nanoparticles [10]. A clear decrease in the relative intensity of the vibrational modes at 1430 cm^−1^, 1500 cm^−1^, and 1540 cm^−1^ can be distinguished, which can be due to structural changes in the polymer related to interactions between the polymer and the SnO nanoparticles. Actually, some authors propose conformational changes in the polymer coils are due to electrostatic interactions with embedded nanoparticles showing charged surfaces [19]. These changes can affect the vibrational bands of specific functional groups, which leads to variations in the Raman signal.

Electrical characterization of the samples was performed by Hall effect measurements. Averaged values of charge carrier concentration, resistivity, and conductivity are shown in Table 1. Bare PEDOT:PSS shows good p-type conductivity with averaged values of 1.9 10^2^ Ω^−1^ cm^−1^, similar to other reported values [37] due to the addition of DMSO and Triton-X-100. Hybrid composite exhibits improved conductivity as compared with the bare polymer, reaching averaged values of about 6.3 10^2^ Ω^−1^ cm^−1^ and high charge carrier concentration values around 1.4 10^22^ cm^−3^, even when the conductivity of SnO nanoparticles shows lower values around 1.8 10^-2^ Ω^−1^ cm^−1^. Actually, conductivity values of about 5·10^-6^ (Ω^−1^ cm^−1^) have been reported for bulk SnO at room temperature [38,39]. Some authors reported that, when the surface of the nanoparticles embedded in the polymer is positively charged, the negatively charged PSS chains can be electrostatically attracted, leading to changes in the PEDOT:PSS chains’ configuration, which can enhance the electrical conductivity [21]. Rearrangement of the PEDOT:PSS can lead to better pathways for charge transport—actually, an expanded-coil configuration is usually associated with improved conductivity [32]. Hence, in this case, synergy between the counterparts in the composite leads to improved electrical performance.

### 2.4. Silicon Surface Passivation

**Figure 6:**
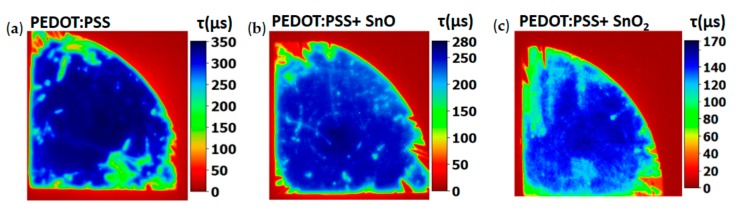
PL images and quasi steady state photoconductance (QSS-PC) lifetime values from: (**a**) PEDOT:PSS; (**b**) hybrid composite; (**c**) results from an analogous hybrid composite using SnO_2_ nanoparticles (1% wt.), included for comparison.

In order to evaluate the optoelectronic applicability of the hybrid composite, its use for Si surface passivation in solar cells was evaluated based on PL imaging and QSS-PC values of charge photocarrier lifetimes. Figure 6 shows PL images from the tested samples, where color bars indicate charge carrier lifetime values (τ) in μs. The corresponding QSS-PC curves under an injection level of 10^15^ cm^−3^ are included as Supplementary Information (Figure S2). Averaged carrier lifetime value around 410 µs was estimated for bare PEDOT:PSS (Figure 6a), while the hybrid composite shows slightly lower, but still good, lifetime value around 308 µs (Figure 6b). Similar measurements were performed in hybrid composites using SnO_2_ nanoparticles (1% wt.) for comparison, leading to lower lifetime values of 137 μs (Figure 6c). In this case, formation of SnO_2_ from SnO should be prevented as worse passivation behavior is achieved by the former.

To summarize, by adding SnO nanoparticles to PEDOT:PSS, improved stability and electrical conductivity, as well as a weak increase in the absorption, are promoted in the hybrid composite which still exhibits good passivation performance. Synergy effects are observed between both organic and inorganic counterparts, as the SnO nanoparticles provide stability and improved optoelectronic response, while the polymer encapsulates SnO nanoparticles, hindering their change into SnO_2_.

## 3. Materials and Methods

SnO nanoparticles were synthesized by a soft chemistry route based on hydrolysis of the starting compound. The selected precursor SnCl_2_ · 2H_2_O (Sigma-Aldrich purity 99.99%, Darmstadt, Germany) was initially dissolved in water. After dissolution, and with continuous stirring at low temperature, NH_4_OH was added until pH = 8 was obtained and hydrolysis occurred. Then, the temperature was increased up to 100 °C for 2 h. The final product was centrifuged and washed several times until reaching neutral pH and dried at 50 °C for 12 h.

For the preparation of the hybrid composite, PEDOT:PSS (Clevios, PH1000, 1.0%–1.3% wt. in water) in solution with the SnO nanoparticles in a controlled concentration (1% wt.) was deposited by spin coating onto silicon n-type float-zone substrates (TOPSIL, thickness 280.00 ± 20 nm) or glass substrates previously treated with isopropanol and boiling water. In order to avoid nanoparticle agglomeration as well as to enhance dispersion and conductivity in the composite, dimethyl sulfoxide (DMSO, Sigma-Aldrich) was added in 5% wt. [34,40]. Triton X-100 was also added in 0.1% wt. in order to achieve improved wettability and slightly increase conductivity [41]. After magnetic stirring for 2 h, the mixture was filtered with a Polyethersulfone (PES) membrane (pore size = 0.45 μm) to achieve higher homogeneity.

Bare PEDOT:PSS films were deposited by spin coating onto Si or glass substrates following different steps. Firstly, an initial step using 2000 rpm for 50 s was used, followed by a second step using 6000 rpm for 20 s, and finally waiting for 10 s for a complete stop. Hybrid composites were deposited following the same procedure as before until the deposition step, in which 1% wt. of the SnO nanoparticles were added to the mixture and mixed in an ultrasonic bath for 15 min and then 30 s with an ultrasonic probe.

Characterization of the samples was carried out by diverse experimental techniques. X-ray diffraction (XRD) in PANanalytical X’Pert Powder equipment (PANanalytical, Malvern, United Kingdom), using the Cu-Kα line where λ_Cu_ = 1.5404 Å. Transmission electron microscopy (TEM) and selected area electron diffraction (SAED) were performed in a Jeol JEM 1400 TEM microscope (Jeol, Japan). Raman spectroscopy measurements were carried out on a Horiba Jobin Yvon LabRam HR 800 using a He-Ne UV laser (λ = 633 nm) and a He-Cd laser (λ = 325 nm). Photoluminescence (PL) was studied at room temperature using the same confocal microscope using a He-Cd UV laser (λ = 325 nm) as the excitation source and charge coupled device (CCD). Different neutral filters were used in order to attenuate the total laser intensity, when necessary. In this configuration, nominal excitation light can be attenuated by using neutral filters to reduce the total laser intensity by a factor of 0.5, 0.25, and 0.1. AFM study was carried out in a Nanotec AFM controlled by Dulcinea electronics, using a silicon tip in contact mode (NanoTec Electrónica, Madrid, Spain). Optical absorption was measured with a UV-vis-NIR light source DH-200 ocean optics with a Deuterium and Halogen lamp. Hall effect measurements were performed at room temperature using a Hall Ecopia AMP55T HMS-7000 with 4 gold probes. Gold contacts around 20 nm were fabricated via Au evaporation on a Quorum Q150T ES using a mask in order to obtain a square for the Van der Pauw configuration (Ecopia, Shanghai, China). On top of the Au contacts, silver paint was added. Finally, the effective charge carrier lifetime values were calculated from the PL intensity based on the quasi steady state photoconductance (QSS-PC) measurements using an LIS-R1 PL imaging setup from BT Imaging with an excitation wavelength of 808 nm and a constant illumination intensity of 4.2 × 10^−2^ W cm^−2^ (BT imaging, Waterloo, Australia).

## 4. Conclusions

SnO nanoparticles were synthesized via hydrolysis, showing high purity and crystallinity. TEM measurements reveal SnO nanoparticles with dimensions around 10 nm, together with a low amount of SnO nanoplates 20–50 nm wide. Rapid transition from SnO to SnO_2_ can be induced by UV irradiation, as demonstrated by Raman spectroscopy and PL. PEDOT:PSS layers were successfully fabricated via spin coating onto Si or glass substrates, obtaining smooth surfaces. Raman spectroscopy indicates changes in the vibrational modes around 1400 cm^−1^ during UV illumination, probably due to variation from benzoid to quinoid form, involving reduced stability of the polymer under irradiation. By adding SnO nanoparticles (1% wt.) to the PEDOT:PSS, enhanced electrical conductivity was achieved (6.3 10^2^ Ω^−1^ cm^−1^), also leading to improved stability of the composite under UV irradiation, and a weakly enhanced optical absorption. Conformational changes in the polymer chains induced by the presence of the embedded nanoparticles can lead to variations in the electrical conductivity. Enhanced conductivity and improved stability is a first-step factor for possible photovoltaic applications.

Finally, Si passivation behavior was evaluated and charge carrier lifetime values of τ~308 µs were obtained for the SnO-based polymer, which is worsened when SnO_2_ nanoparticles are embedded in the composite.

## Figures and Tables

**Table 1:** Hall effect measurements performed with I = 0.1 mA.

Sample	Charge Carrier Concentration (cm^−3^)	Resistivity (Ω cm)	Conductivity σ (Ω^−1^ cm^−1^)
SnO	(2.98 ± 0.66)·10^15^	(5.49 ± 0.12)·10^1^	(1.82 ± 0.21)·10^−2^
PEDOT:PSS	(7.37 ± 1.38)·10^21^	(5.30 ± 0.03)·10^−3^	(1.89 ± 0.01)·10^2^
Composite	(1.41 ± 0.27)·10^22^	(1.58 ± 0.02)·10^−3^	(6.33 ± 0.01)·10^2^

## Supplementary Materials

The following are available online.
