# Supplementary material for: Synergetic Improvement of Stability and Conductivity of Hybrid Composites formed by PEDOT:PSS and SnO Nanoparticles
Source: Molecules. 2020 Feb 6;25(3):695. doi: 10.3390/molecules25030695 (PMC7036770; doi:10.3390/molecules25030695)
Supplement: Supplementary file 1 [file molecules-25-00695-s001.pdf]

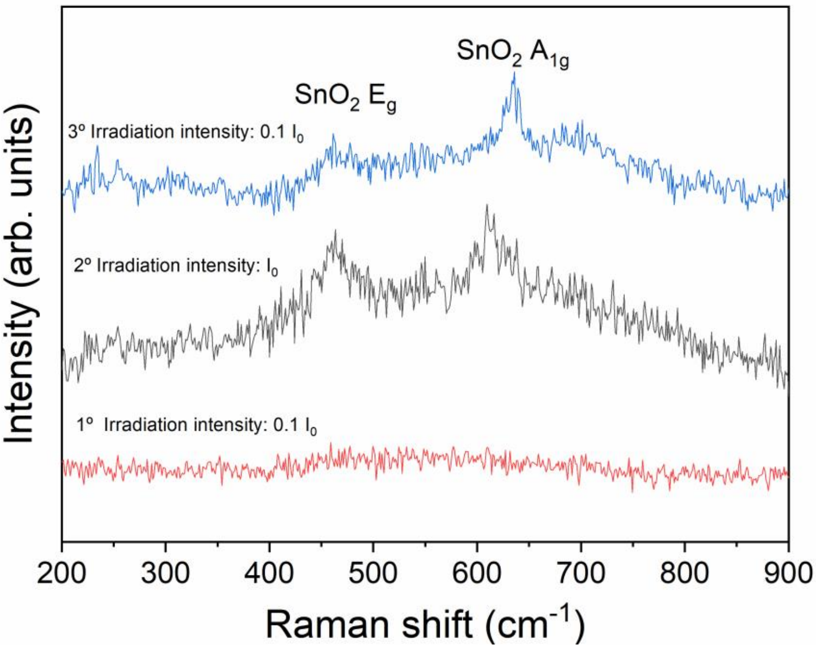

Figure S1. Raman spectra of the laser-induced transition from SnO to SnO_2. Initially, no modes corresponding to SnO_2 are observed with a laser power of 0.1 I_0. Using a power density corresponding to I_0, SnO_2 modes E_g and A_{1g} are detected. This transition is not reversible, as SnO_2 modes remain after subsequent irradiation with the lower laser power 0.1 I_0.

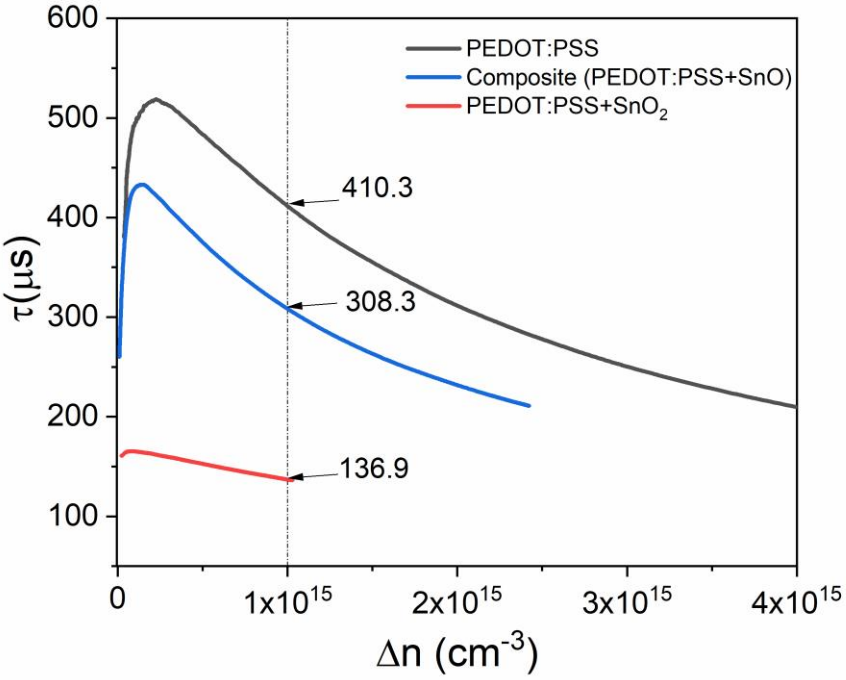

Figure S2. QSS-PC measurements corresponding to the samples shown in Figure 6.
